# Homologous recombination-mediated cloning and manipulation of genomic DNA regions using Gateway and recombineering systems

**DOI:** 10.1186/1472-6750-8-88

**Published:** 2008-11-17

**Authors:** Kevin Rozwadowski, Wen Yang, Sateesh Kagale

**Affiliations:** 1 Saskatoon Research Centre, Agriculture and Agri-Food Canada, 107 Science Place, Saskatoon, Saskatchewan, Canada, S7N 0X2

## Abstract

**Background:**

Employing genomic DNA clones to characterise gene attributes has several advantages over the use of cDNA clones, including the presence of native transcription and translation regulatory sequences as well as a representation of the complete repertoire of potential splice variants encoded by the gene. However, working with genomic DNA clones has traditionally been tedious due to their large size relative to cDNA clones and the presence, absence or position of particular restriction enzyme sites that may complicate conventional *in vitro *cloning procedures.

**Results:**

To enable efficient cloning and manipulation of genomic DNA fragments for the purposes of gene expression and reporter-gene studies we have combined aspects of the Gateway system and a bacteriophage-based homologous recombination (i.e. recombineering) system. To apply the method for characterising plant genes we developed novel Gateway and plant transformation vectors that are of small size and incorporate selectable markers which enable efficient identification of recombinant clones. We demonstrate that the genomic coding region of a gene can be directly cloned into a Gateway Entry vector by recombineering enabling its subsequent transfer to Gateway Expression vectors. We also demonstrate how the coding and regulatory regions of a gene can be directly cloned into a plant transformation vector by recombineering. This construct was then rapidly converted into a novel Gateway Expression vector incorporating cognate 5' and 3' regulatory regions by using recombineering to replace the intervening coding region with the Gateway Destination cassette. Such expression vectors can be applied to characterise gene regulatory regions through development of reporter-gene fusions, using the Gateway Entry clones of GUS and GFP described here, or for ectopic expression of a coding region cloned into a Gateway Entry vector. We exemplify the utility of this approach with the Arabidopsis *PAP85 *gene and demonstrate that the expression profile of a *PAP85::GUS *transgene highly corresponds with native *PAP85 *expression.

**Conclusion:**

We describe a novel combination of the favourable attributes of the Gateway and recombineering systems to enable efficient cloning and manipulation of genomic DNA clones for more effective characterisation of gene function. Although the system and plasmid vectors described here were developed for applications in plants, the general approach is broadly applicable to gene characterisation studies in many biological systems.

## Background

Genes are typically characterised by both assessing the phenotypic effects of the encoded gene product through its over- or ectopic-expression, and by detailing the temporal and spatial patterns of expression of the gene by linking its regulatory regions to a reporter gene. Characterising gene products has been greatly facilitated by the availability of cloned full-length cDNAs and open-reading frames (ORFs) from public stock centres and commercial suppliers. These collections provide significant time- and cost-savings by obviating the need to independently clone cDNAs for genes of interest; however, reliance on existing cDNA collections and the use of cDNA clones exclusively in gene characterisation studies has several limitations. Firstly, current collections are not fully comprehensive, a consequence of the technical challenges of getting complete representation of the ORFeome from all tissue types under all possible environmental conditions and physiological and developmental states. Furthermore, certain transcripts may be low in abundance or may be of a size or sequence composition that inhibits cloning of full-length cDNAs. As a result, many predicted genes identified by analysis of genome sequences are not represented in current cDNA collections or are represented only by partial clones. Secondly, assignment of gene functions based on the phenotypic effects caused by expression of a cDNA may not accurately represent the phenotypic potential of the gene due to the possible regulatory effects of non-coding regions of the native gene. Indeed, introns and untranslated regions (UTRs) have been shown to encode small regulatory RNAs or their binding-sites as well as the recognition sequences for RNA-binding proteins which affect turnover and translation rates of mRNA transcripts and their subcellular localisation [1-3]. In addition, intron processing and structural properties of UTRs can affect mRNA accumulation, translation, nuclear export and subcellular targeting [4-7]. With respect to the influence of UTRs, the translation efficiency and tissue specificity of reporter genes in transgenic plants can be directly affected by the type of UTR, as shown using the UTRs from the small subunit of rubisco from amaranth and *Flaveria bidentis *[8,9]. In addition, the 3' UTR of soybean glutamine synthetase functions in transcript turnover and translation repression in both the leaves and nodules of transgenic alfalfa [10]. The involvement of the 5' UTR of the maize alcohol dehydrogenase 1 gene in enhancing translation under stress conditions has also been demonstrated [11]. The influence of introns on gene expression has been shown using the first intron of the actin-depolymerising factor 1 gene of petunia and Arabidopsis which can alter expression pattern and tissue specificity by a post-transcriptional mechanism [12]. There is also growing understanding of the diversity of gene products and phenotypic effects attributable to alternative splicing of pre-mRNAs [13-20]. Thus accurate interpretation of gene function can only be effectively obtained using genomic DNA (gDNA) clones comprising entire coding regions in expression studies with subsequent analysis of individual splice variants using corresponding cDNAs. Unfortunately, the routine use of gDNA clones has been impeded by difficulties associated with their large size and its effect on cloning efficiency, as well as the availability and position of unique restriction enzyme sites to enable conventional cloning procedures.

The technical difficulties of working with gDNA fragments have also impeded efforts to characterise gene regulatory regions. Typically, only the 5 ' regulatory region (promoter) is assessed in such investigations by creating transcriptional fusions to a reporter gene (e.g. GUS, LacZ, GFP) that is linked to a generic transcription terminator signal. This approach has been generally informative but may not result in the most accurate representation of gene expression patterns due to the use of non-cognate transcription terminators and 3' UTR sequences that may lack important regulatory signals. Therefore, the use of both the cognate 5' and 3' regulatory regions in expression studies using reporter genes is essential.

The time requirements and limitations of traditional *in vitro *cloning procedures have been significantly reduced through the development of the Gateway cloning system [21] and recombineering cloning [22]. The Gateway system uses elements of the site-specific recombination system of bacteriophage lambda to enable inter-molecular transfer of DNA fragments between vectors. Assembly of Gateway Entry clones of DNA fragments depends upon conventional *in vitro *cloning procedures. This system has been employed in large-scale cDNA projects and compatible vectors have been developed for applications in many systems including plants where Gateway vectors are available for the analysis of plant gene functions by ectopic overexpression, promoter-reporter fusions, affinity purification tags, RNA interference, complementation analysis and gene stacking [23-26]. Recombineering exploits homologous recombination functions in *Escherichia coli*, such as the Red recombination proteins of bacteriophage lambda, to enable cloning and modification of DNA fragments *in vivo*. This system has been used to subclone or "rescue" gDNA fragments as large as 80 kb from a bacterial artificial chromosome (BAC) into a standard plasmid vector [27]. Recombineering is a powerful technique because it enables cloning of DNA fragments independent of restriction enzymes or PCR-based manipulation of the DNA fragment to be cloned. A significant limitation of recombineering is that transfer of DNA fragments between different vectors is not as efficient as for the Gateway system.

Here we describe a system that combines features of the Gateway and recombineering techniques. We demonstrate that a genomic coding region can be sub-cloned into a Gateway Entry vector from a BAC using recombineering. We also show how Gateway-compatible expression vectors that incorporate cognate 5' and 3' regulatory regions of a gene of interest can be created by using recombineering to substitute the coding region of a gene with Gateway recombination sites. By incorporating the GUS reporter gene adjacent to the regulatory regions of the Arabidopsis *PAP85 *gene we demonstrate this system enables accurate representation of gene expression patterns. The synergy in efficiency of gene cloning and manipulations achievable by combining Gateway and recombineering techniques should greatly expedite gene characterisation studies at the single-gene and large-scale, genome-wide levels.

## Results and discussion

### Cloning of genomic coding regions

To illustrate the direct construction of Gateway-based Entry clones encoding genomic coding regions by recombineering we selected the Arabidopsis *ABI3 *gene. *ABI3 *encodes a BY-domain containing transcription factor that plays a central role in embryo and seed development as well as in regulating physiological responses to abiotic stress [28,29]. Orthologues of *ABI3 *with similar roles have been characterised in a variety of crop and tree species [29-31]. *ABI3 *orthologues in wheat have been shown to produce at least seven different transcripts by alternative splicing [32] and splice variants of an *ABI3 *homologue from *Brassica napus*, a close relative of Arabidopsis, have also been detected (K. Rozwadowski, unpublished). Thus, to characterise the effects of overexpressing a particular gene in plants, the use of a single full-length cDNA variant may not be the most informative as it will not represent the complete transcriptional repertoire of the gene and may incompletely reflect gene function.

**Figure 1:**
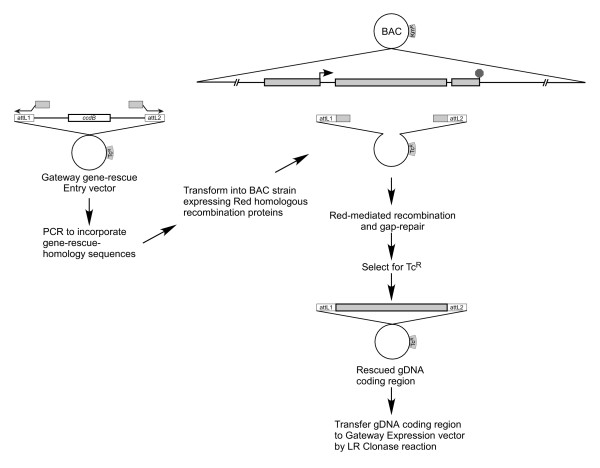
**Rescue of genomic coding region into a Gateway Entry vector**. The Entry vector is amplified by PCR using primers that incorporate 50 bp of homology corresponding to the 5' and 3' ends of the coding region for the gene of interest. The vector amplicon is transformed into an *E. coli *strain possessing a BAC containing the gene of interest and expressing the bacteriophage lambda Red homologous recombination proteins. Selection for the Entry vector identifies clones containing the desired genomic coding region.

The overall approach to cloning or "rescuing" gDNA fragments from a BAC to create a Gateway Entry clone is outlined in Figure 1. A BAC encoding the gene of interest is first transformed into *E. coli *EL250, a strain deficient for host recombination functions to help ensure stability of the BAC clone and which encodes the Red homologous recombination functions of bacteriophage lambda under control of an inducible promoter [27]. The rescue-vector into which the gene sequence of interest is to be subcloned from the BAC is then manipulated by PCR to incorporate sequences homologous to the 5' and 3' ends of the desired gene sequence. These "rescue-homology" sequences are 50 bp in length and are linked to "anchor sequences" which are unique to the rescue-vector. The rescue-homology sequences and anchor sequences should not possess homology to one another to prevent any recombination between the ends of the rescue-vector amplicon. In this system the rescue-vector is an Entry vector of the Gateway system that incorporates the phage lambda attL1 and attL2 site-specific recombination sites. The rescue-vector amplicon encoding the 5' and 3' rescue-homology sequences is then transformed into competent cells containing the target BAC and the Red recombination proteins. A recombination event involving gap-repair occurs between the ends of the rescue vector amplicon and the homologous regions flanking the desired gene encoded by the BAC clone. These events are selected for by using a unique selectable marker on the rescue-vector. Thus the system allows for direct construction of a Gateway Entry clone of a gDNA subclone from a BAC.

The BAC clone F22P10 encoding *ABI3 *is based on the pBeloBAC-Km vector backbone [33] which encodes the kanamycin-resistance (Km^R^) bacterial selectable marker. The pENTR Gateway vector series also utilise the Km^R ^marker thus preventing direct selection of genomic fragments rescued from the BAC. In addition, published Gateway-based plant transformation vectors [23,24] also primarily use the Km^R ^selectable marker, again precluding selection of recombinants resulting from gene transfer from Entry vectors to Destination vectors. Therefore, we created a pENTR2B derivative, pJM1, whereby the vector Km^R ^marker is inactivated by insertion of a tetracycline-resistance (Tc^R^) marker. Tetracycline resistance is not used in common BAC or plant transformation vectors and thus pJM1 should have broad applicability for rescuing gDNA fragments from BAC clones and enabling their efficient Gateway-based transfer to a variety of expression vectors.

**Figure 2:**
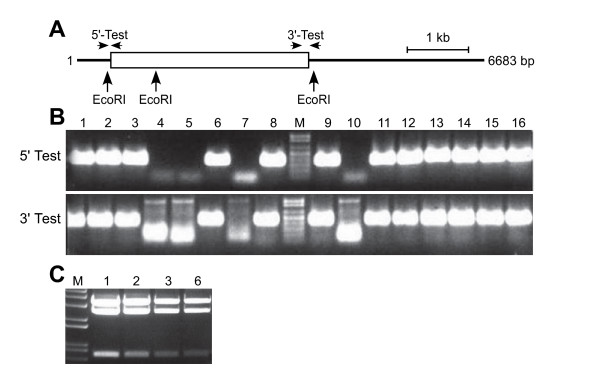
**Rescue of the *ABI3 *coding region into a Gateway Entry vector**. (A) Schematic diagram showing the location of primers and restriction enzyme sites used for analysis of the integrity of candidate clones of pWY148 encoding *ABI3 *in pJM1. Boxed region represents the *ABI3 *genomic coding region, and the line represents vector sequence. Arrows indicate location of diagnostic PCR primers and EcoRI sites used for evaluating the correctness of the assembled construct. (B) PCR-based screening of clones potentially encoding *ABI3 *in pJM1. The 5'-Test (primers Entry-L1 and ABI3-5'-CDS-Rescue-3'-Test) and 3'-Test (primers Entry-L2 and ABI3-3'-Term-Rescue-5'-Test) evaluates for the fusion of the vector with the 5' or 3' end of the *ABI3 *coding region. An amplicon of 0.4 kb or 0.5 kb is expected for correct vector-gene fusions at the 5' or 3' end, respectively. (C) Restriction enzyme analysis of candidate *ABI3 *clones in pJM1. Clones 1, 2, 3 and 6 from panel A were digested with EcoRI and exhibit the predicted restriction fragments of 0.8, 2.6 and 3.4 kb. M, DNA size marker (1 kb Plus ladder, Invitrogen) with representation from 0.1–1 kb (B) or 0.65–5 kb (C).

To rescue the *ABI3 *coding region from BAC F22P10, pJM1 was amplified by PCR using the primers ABI3-L1-Rescue-5'CDS and ABI3-L2-Rescue-3'CDS (the sequences of these and all other primers used in this study are listed in Table 1) to incorporate the homology regions required to rescue the *ABI3 *coding region from BAC F22P10 into the vector. The unique anchor sequences for these primers in pJM1 were chosen to maintain the restriction sites in the multiple cloning site to enable future manipulation of the rescued gDNA sequence by conventional methods, if required. The 5' rescue-homology region encodes 50 bp starting 12 bp upstream of the *ABI3 *translation start codon, whereas the 3' rescue-homology region encompasses the sequence 329–379 bp 3' of the translation stop codon thereby including the entire 3' UTR and predicted transcription termination signals of *ABI3 *based on the annotated Arabidopsis genome sequence () [34]. The rescued fragment encoding *ABI3 *is predicted to be 3262 bp. Despite the size and complexity of the *ABI3*-rescue primers PCR amplification of the 3.8 kb pJM1 was robust under standard conditions (data not shown). The pJM1 rescue-vector amplicon was then transformed into competent *E. coli *EL25/F22P10 cells induced to express Red recombinase proteins and candidate clones were selected in the presence of tetracycline. From several hundred tetracycline-resistant colonies sixteen were screened by PCR to assess if the *ABI3 *gene was rescued into pJM1. The primer pairs were Entry-L1 with ABI3-5'-CDS-Rescue-3'-Test, and Entry-L2 with ABI3-3'-Term-Rescue-5'-Test combining a vector-specific with a gene-specific primer to assess if the 5' and 3' regions, respectively, of *ABI3 *were present in candidate clones. As shown in Figure 2, PCR tests for 12 of 16 clones resulted in the predicted amplicons of 0.4 kb and 0.5 kb corresponding to the 5' and 3' regions, respectively, of the rescued *ABI3 *coding region. Four of the rescued *ABI3 *clones were assessed by restriction enzyme digests using EcoRI and all possessed the predicted fragments of 0.8 and 2.6 kb representing *ABI3*, and 3.4 kb representing pJM1 (Figure 2). One representative of the four clones was sequenced and shown to encode the sequence of the *ABI3 *coding region predicted to be subcloned. This clone was designated pWY148.

**Table 1:** Oligonucleotide primers used in this study

**Primer name**	**Sequence 5'-3'**
Tc-5'-PmlNco	CACGTGCCATGGTTCTCATGTTTGACAGCTTATC
Tc-3'-PmlNsi	CACGTGAT GCATGGACTTCCGCGTTTCCAG
SpR-5'-HpaBspNhe	ATCGTTAACTCATGAGCTAGCCGTTCGTGAATACATGTTATAATA
SpR-3'-Hpa	ATCGTTAACTTATTTGCCGACTACCTTGGTGATCT
GFP-mu-5'-Bam	ATCGGATCCAAAACAATGGCTAGTAAAGGAGAAGAACTTTTCACTGGAGTTGTCCC
GFP-mu-3'-NotAvrBgl	GATATCGCGGCCGCCCTAGGTAGATCTTCCACCTCCACCTTTGTATAGTTCATCCATGCCATGTGTA
ABI3-5'-CDS-Rescue-3'-Test	CTCCTCCATGGTCATCTAACC
ABI3-3'-CDS-Rescue-5'-Test	GTACCCGAGTGTGCCACTTC
PAP85-Prom-Rescue-3'-Test	ACTTCCTAGGGTTGCTACTG
PAP85-Term-Rescue-5'-Test	ACCGGATGGTTTGGT TATGG
ABI3-L1-Rescue-5'-CDS^1^	agatctccggcgttggccgccacatgcaagcttttcatcgttgaagtggaCAAATACGCATACTGTTATCTGGCT
ABI3-L2-Rescue-3'-CDS^1^	tattacatatgtggctcatttcttgagtcgagcttacatatcgtttataaAACCTGATGTTCTGGGGAATATAG
ENTRY-L1	TTAGTTAGTTACTTAAGCTCGG
ENTRY-L2	TGAGACACGGGCCAGAGC
PAP85-Rescue-5'-Prom^2^	ttcctaccaccatgaccaaacttggaatcccgaaactccctcttcttgttTTGTCGTTTCCCGCCTTCAG
PAP85-Rescue-3'-Term^2^	atagctcgtattttttagcttatgtctaaaatacccctgagatcatgtggAACGTTCTCGAGGGGATCTTCTG
pCB-RB	CTGTGGTTGGCATGCACATA
tCUP2-Out	CGAGGTTAGACCTTCAGGAAATA
PAP85-Integ-Dest-5'^3^	acccattattgtcctcaaaaacaaacacatcaacaaaacaacaagaaaaaGATATCACAAGTTTGTACAAAAAAGC
PAP85-Integ-Dest-3'^3^	ttaaactcttaaactctttcctttcttaggactgtaaaaaagcattttcaGATATCACCACTTTGTACAAGAAAGC
PAP85-Dest-Integ-5'-Test	TGATGCGTACGAACACTC
Cm-5'-AS	ACGGTGGTATATCCAGTGATT
PAP85-Dest-Integ-3'-Test	GTTACATGCAGAGCG TTACC
C1	TTATACGCAAGGCGACAAGG
PAP85-S1	GCAGGGATCCT TGTTCCTTC

^1^Upper-case letters correspond to unique recognition sequences in pJM1 and lower-case letters correspond to 50 bp regions homologous to *ABI3*.

^2^Upper-case letters correspond to unique recognition sequences in pWY109 and pWY107 and lower-case letters correspond to 50 bp regions homologous to *PAP85*.

^3^Upper-case letters correspond to unique recognition sequences in the RfA-Destination cassette of pMW138-1 and lower-case letters correspond to 50 bp regions homologous to *PAP85*.

This approach for cloning gDNA coding regions takes only 2 days from the initial transformation of the *E. coli *EL250 BAC strain with the rescue-vector amplicon to confirmation of the candidate clones by PCR. The primers used to incorporate rescue-homology were large but did not require purification so overall expense was minimal. Although the vector is amplified by PCR using a high-fidelity polymerase, mutation(s) could occur during the amplification; however, the cloning system directly selects for function of essential vector components (i.e. selectable marker, origin of replication). The *in vivo *gap repair mechanism for rescuing the specified fragment involves the host *E. coli *DNA replication and proofreading machinery; thus the sequence fidelity of the rescued fragment should routinely be very high. Therefore using this system, no cost or delay is typically required for sequencing the clone as is generally required when a PCR step is used to incorporate restriction enzyme sites to enable conventional cloning of a DNA fragment. The system is also independent of the requirement for full-length cDNA clones to enable expression of genes and circumvents the need to clone and express individual splice-variants to assess functions of a gene.

### Cloning of coding regions and cognate regulatory sequences for construction of Gateway-based expression vectors

**Figure 3:**
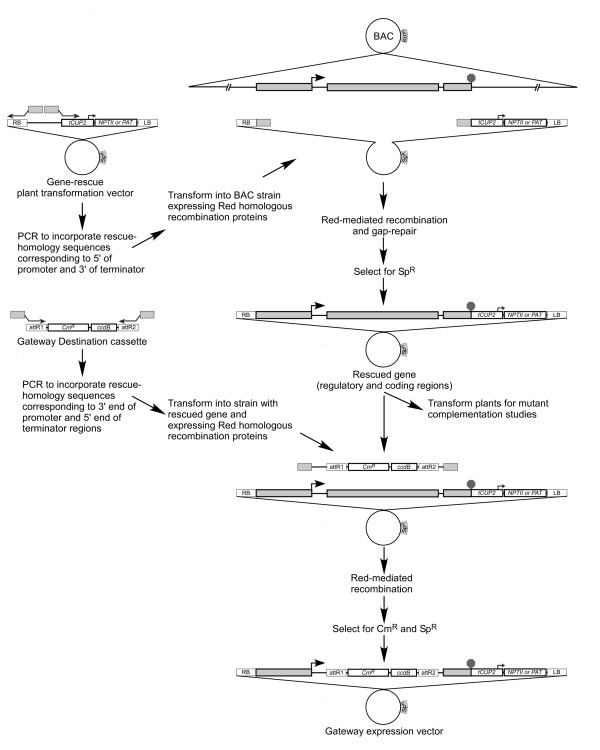
**Rescue of regulatory and coding regions directly into a plant transformation vector and development of a Gateway Expression vector**. To rescue the regulatory and coding regions of a gene, the plant transformation vector is amplified by PCR using primers that incorporate 50 bp of homology corresponding to sequences 5' and 3' of the promoter and terminator regions. The vector amplicon is transformed into an *E. coli *strain possessing a BAC containing the genomic region of interest and expressing the bacteriophage lambda Red homologous recombination proteins. Selection for the plant transformation vector identifies clones containing the rescued gene which can be directly used to transform plants using *A. tumefaciens*. To create a novel Gateway Expression vector incorporating the 5' and 3' regulatory regions of the rescued gene, the Gateway Destination cassette is first amplified by PCR using primers that incorporate 50 bp of homology corresponding to sequences 3' of the promoter and 5' of the terminator regions. The Destination cassette amplicon is transformed into an *E. coli *strain possessing the cloned gene in a plant transformation vector and expressing the bacteriophage lambda Red homologous recombination proteins. Selection for the plant transformation vector and the marker on the Destination cassette identifies clones where the original cloned coding region is replaced by the Destination cassette. The Destination cassette now flanked by the cognate 5' and 3' regulatory regions of the initial cloned gene can be used to link reporter genes or other genes of interest to the cloned regulatory regions.

Cloning of gDNA fragments including the coding region and cognate regulatory elements of a gene of interest is often required in gene identification and characterisation studies. For example, in mutant complementation studies, more accurate representation of the native gene expression level achievable by using genomic clones is beneficial to identifying the correct gene; in contrast, using a candidate cDNA linked to a constitutive promoter may cause pleiotropic effects that can confound interpreting complementation results. Assessing the temporal and spatial expression patterns of a gene by linking its regulatory regions to reporter genes is also a central component of gene characterisation. A system for direct cloning of gDNA fragments from BACs into a plant transformation vector and creating expression constructs for reporter gene fusions by recombineering is outlined in Figure 3. The process is very similar to that described above for rescuing gene coding regions into a Gateway Entry vector (Figure 1). The distinction here is that the gene including its 5' and 3' regulatory sequences are rescued directly into a plant transformation vector to expedite assembly of a plant transformation construct. This construct can then be directly converted to a Gateway-based expression construct by substituting the protein encoding region of the cloned gene for a cassette containing site-specific recombination sites (i.e. the Gateway Destination cassette). In this way, reporter or other genes may be efficiently linked to both the 5' and 3' regulatory regions. Analysis of transgenic lines using such a reporter construct is anticipated to more faithfully represent the expression pattern of a gene than if a generic 3' regulatory region is used.

Subcloning of gDNA regions from BACs by recombineering requires PCR amplification of the plant transformation vector to incorporate the 50 bp of 5' and 3' rescue-homology regions. The majority of existing plant transformation vectors are quite large (typically 10 kb or more) and therefore may not amplify well using PCR. In addition, plant transformation vectors typically use Km^R ^selection in bacteria which is problematic for the gene rescue system developed here since many plant BAC libraries also use the same selection. We thus developed compact plant transformation vectors with unique features for use in our gene-rescue system. The base vector was pCB301 which is a 3.5 kb derivative of pBin19 created by combining the minimal essential elements required for a binary plant transformation vector [35]. The Km^R ^marker of pCB301 was inactivated by insertion of the *aad9 *gene from *Enterococcus faecalis *which confers spectinomycin-resistance (Sp^R^) to *E. coli *and *Agrobacterium tumefaciens*. The resulting construct was designated pNML54. The Sp^R ^marker is compatible with the selection used for BAC vectors, and is different from the selection used for existing Gateway Entry vectors which employ Km^R ^selection. pNML54 was modified to create the gene-rescue vectors pWY107 and pWY109 which incorporate either the *NPTII *or *PAT *gene, respectively, within the T-DNA region to function as selectable markers in plants. This pair of gene-rescue vectors provide options for selection when experiments require complementation of T-DNA insertion-mutant alleles of Arabidopsis which may already possess Km^R ^or phosphinothricin-resistance due to the presence of the *NPTII *or *PAT *genes, respectively, in the mutagenic T-DNA cassette ([36-38]; I. Parkin, unpublished). The plant selectable markers of pWY107 and pWY109 are linked to the EntCUP2 promoter which is active in Arabidopsis and several other dicot species [39]. Use of this unique promoter should minimise interference with the expression of selectable markers or activation-tagged genes resident in Arabidopsis T-DNA insertion lines which typically employ the CaMV35S promoter ([36-38]; I. Parkin, unpublished).

**Figure 4:**
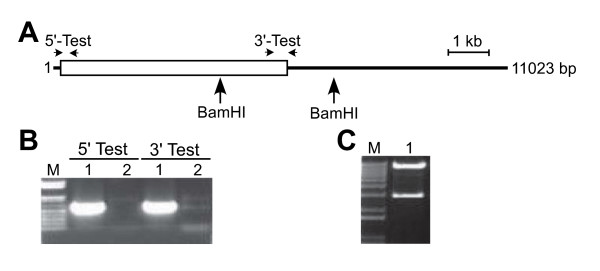
**Rescue of *PAP85 *regulatory and coding regions into a plant transformation vector**. (A) Schematic diagram showing the location of primers and restriction enzyme sites used for analysis of the integrity of candidate clones of pWY189 encoding *PAP85 *in pWY109. Boxed region represents *PAP85 *regulatory and genomic coding regions, and the line represents vector sequence. Arrows indicate location of diagnostic PCR primers and BamHI sites used for evaluating the correctness of the assembled construct. (B) PCR-based screening of clones potentially encoding *PAP85 *in pWY109. The 5'-Test (primers pCB-RB and PAP85-Prom-Rescue-3'-Test) and 3'-Test (primers tCUP2-OUT and PAP85-Term-Rescue-5'-Test) evaluates for the fusion of the vector with the 5' or 3' regulatory regions, respectively. An amplicon of 0.6 kb is expected for correct vector-gene fusion at both the 5' and 3' end. (C) Restriction enzyme analysis of candidate *PAP85 *clones in pWY109. Clone 1 from panel A was digested with BamHI and exhibited the predicted restriction fragments of 2.7 and 8.3 kb. M, DNA size marker (1 kb Plus ladder, Invitrogen) with representation from 0.1–1 kb (B) or 1–12 kb (C).

To illustrate the direct rescue of a gene and its regulatory regions into a plant transformation vector and subsequent conversion to a Gateway-compatible expression vector we selected the Arabidopsis *PAP85 *gene which encodes a vicilin-like seed storage protein [40]. BAC F18C5 (Km^R^) encoding *PAP85 *was transformed into *E. coli *EL250. The rescue-vector pWY109 was amplified by PCR using the primers PAP85-Rescue-5'-Prom and PAP85-Rescue-3'-Term to incorporate the 50 bp homology regions to rescue *PAP85 *from the BAC. These primers encode unique anchor sequences common to both pWY109 and pWY107. The rescue-homology regions correspond to sequences 2500 bp 5' of the translation start codon of *PAP85 *and 1019 bp 3' of the translation stop codon resulting in a predicted rescued fragment of 5498 bp. The pWY109 amplicon was transformed into *E. coli *EL250/F18C5 competent cells induced to express the Red recombination proteins and candidate clones were selected using spectinomycin. Three colonies were obtained and tested by PCR for the presence of the rescued *PAP85 *fragment in pWY109 using the vector-specific primers pCB-RB and tCUP2-OUT in combination with the gene-specific primers PAP85-Prom-Rescue-3'-Test and PAP85-Term-Rescue-5'-Test, respectively. Two clones were positive for the PCR test and produced the 0.6 kb amplicons corresponding to the 5' and 3' ends, respectively, of the rescued gene in pWY109 (Figure 4). One clone was characterised by restriction enzyme digestion using BamHI and was found to contain the predicted 8.3 and 2.7 Kb fragments. The clone was designated pWY189.

This approach to subclone gDNA fragments directly into a plant vector can be accomplished in two days, requires little hands-on time and is completely independent of the need for unique restriction sites in the vector or sequence to be cloned. While the region encoding *PAP85 *described here was 5.5 kb, sequences of up to 45 kb have also been cloned using this system (data not shown). Therefore this system is flexible enough to enable subcloning of small to large genomic fragments.

**Figure 5:**
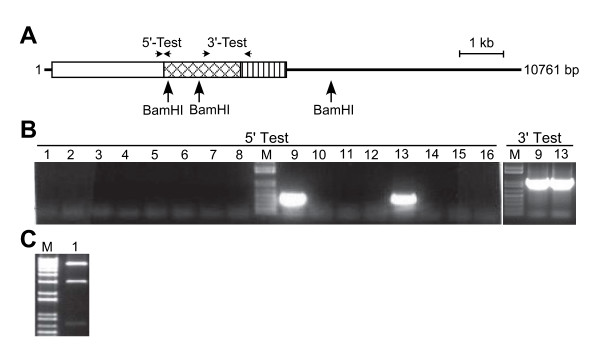
**Insertion of Gateway Destination cassette between rescued regulatory regions of *PAP85***. (A) Schematic diagram showing the location of primers and restriction enzyme sites used for analysis of integrity of candidate clones of pWY190 with the Rfa-cassette replacing the coding region of *PAP85*. Open-boxed region represents 5'- and 3'-regulatory region of *PAP85*, hatched-box region represents the Gateway Destination cassette, vertical striped-box region represents the 3'-regulatory region of *PAP85 *and the line represents vector sequence. Arrows indicate location of diagnostic PCR primers and BamHI sites used for evaluating the correctness of the assembled construct. (B) PCR-based evaluation of candidate clones of pWY190. The 5'-Test (primers Cm-5'-AS1 and PAP85-DEST-Integ-5'-Test) evaluates for correct fusion of attR1 with the 5' regulatory region of *PAP85 *with the presence of a 0.3 kb amplicon. Of 24 candidate clones assessed 16 are represented. The 3'-Test (primers C1 and PAP85-DEST-Integ-3'-Test) evaluates clones 9 and 13 for correct fusion of attR2 with the 3' regulatory region of *PAP85 *with the presence of a 1.1 kb amplicon. (C) Restriction enzyme analysis of candidate *PAP85*-Rfa fusion. pWY190 was digested with BamHI and exhibited the predicted restriction fragments of 0.7, 3.0 and 7.1 kb. M, DNA size marker (1 kb Plus ladder, Invitrogen) with representation from 0.1–1 kb (B) and 0.5–12 kb (C).

To illustrate development of Gateway expression vectors encoding cognate 5' and 3' regulatory regions, the coding region of *PAP85 *in pWY189 was replaced with the Gateway RfA-Destination cassette using recombineering. The RfA-Destination cassette encodes a chloramphenicol-resistance (Cm^R^) marker and the *ccdB *gene which is toxic to most laboratory strains of *E. coli*; therefore, the strain DB3.1 was used in all subsequent manipulations. *E. coli *DB3.1 was co-transformed with pWY189 and pKD46 which encodes the bacteriophage lambda Red homologous recombination functions regulated by arabinose induction and possesses a temperature sensitive origin of replication [41]. The RfA-Destination cassette was amplified by PCR using the primers PAP85-Integ-DEST-5' and PAP85-Integ-DEST-3' which possess anchor sequences unique to the attR1 and attR2 sites of the cassette and 50 bp that is homologous to the sequence immediately 5' or 3' of the translation start and stop codon of *PAP85*, respectively. The RfA-Destination cassette amplicon was transformed into *E. coli *DB3.1/pWY189/pKD46 induced to express the Red homologous recombination proteins and candidate clones selected in the presence of chloramphenicol and spectinomycin. Several hundred colonies were obtained and candidate clones were then screened by PCR for correct integration of the RfA-Destination cassette into pWY189 using the cassette-specific primers Cm-5'-AS1 and C1 in combination with the gene-specific primers PAP85-DEST-Integ-5'-Test and PAP85-DEST-Integ-3'-Test, respectively. Two of 24 candidates were found to be correct as indicated by the amplification of a 0.3 kb fragment representing the fusion between the *PAP85 *promoter and the attR1 end of RfA-Destination cassette and a 1.1 kb fragment representing the fusion between the *PAP85 *terminator and the attR2 end (Figure 5). One clone was designated pWY190. Restriction analysis indicated that the strain containing pWY190 also retained the parent plasmid pWY189 (data not shown). This is likely due to the stability of the R112 origin of replication present on these plasmids. To eliminate pWY189 and to prepare a strain containing only pWY190, a plasmid DNA preparation was digested with BlpI whose recognition site is unique to the *PAP85 *coding region and is thus unique to pWY189. The mixture was then transformed into DB3.1 and clones selected in the presence of spectinomycin. Fifteen colonies were screened by PCR for the presence of pWY190 using the primers PAP85-DEST-Integ-3'-Test and C1 and the absence of pWY189 using the primers PAP85-DEST-Integ-3'-Test and PAP85-S1. Eight isolates were found to possess only pWY190 (data not shown). Three of these were examined by restriction digestion using BamHI and all possessed the expected restriction fragments of 0.7, 3.0 and 7.1 kb (one representative is shown in Figure 5). pWY190 was sequenced and found to contain the exact predicted sequence of the 5' and 3' regulatory regions of *PAP85 *fused to the RfA-Destination cassette. Thus, our system represents a rapid method for developing expression constructs that are Gateway compatible. A distinct feature of this system is the ability to easily maintain both the 5' and 3' regulatory elements of a gene in the expression construct to help ensure that reporter-gene experiments more accurately reflect the expression properties of the gene under study.

### Application of recombineering-Gateway system to characterising gene expression profiles using promoter-reporter transgene constructs

To enable reporter gene studies with this system we constructed a *PAP85::GUS *reporter construct designated as pSW27. This construct was used to transform Arabidopsis and transgenic plants were analysed for GUS expression to assess tissue specificity and developmental regulation of *PAP85*.

**Figure 6:**
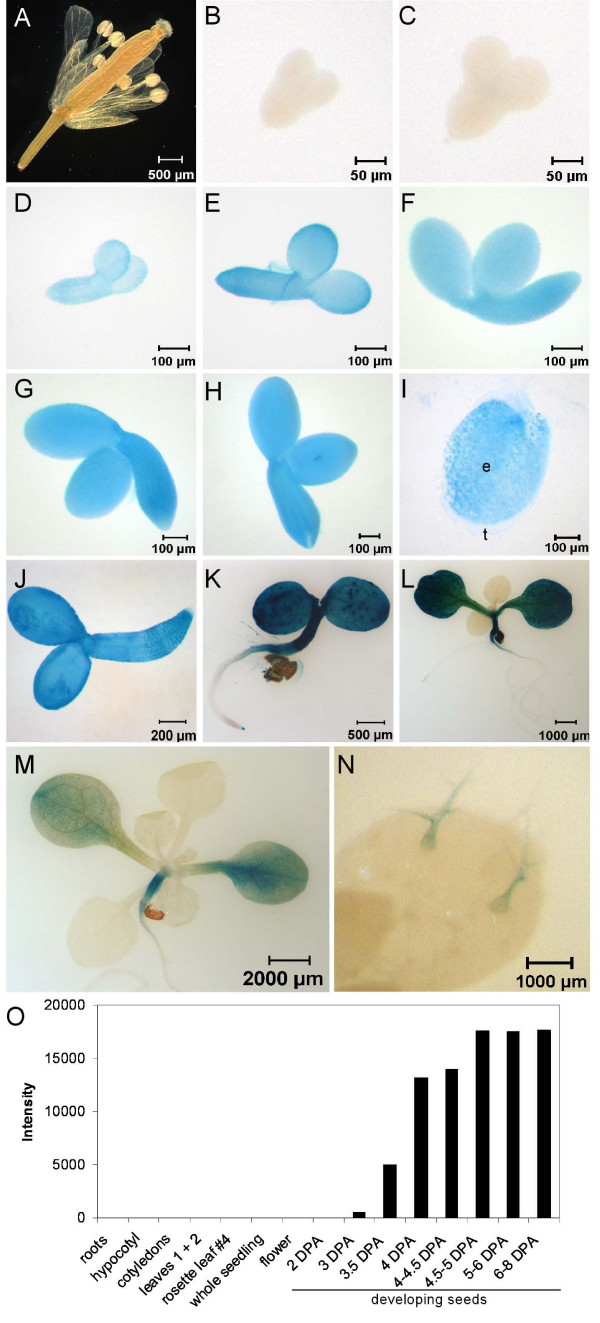
**Expression of *PAP85 *in Arabidopsis as represented by histochemical localization of GUS activity**. Various tissues of a transgenic Arabidopsis line encoding the *PAP85::GUS *reporter construct pSW27 were stained for GUS activity. (A) Flower at one day post-anthesis. (B-H) Developing embryos dissected from siliques collected at 3 (B), 4 (C), 5 (D), 6 (E), 8 (F), 10 (G), and 12 (H) days post-anthesis. (I) Endosperm (e) and testa (t) from developing seed collected at 8 DPA. (J-M) Seedlings collected at 1 (J), 3 (K), 6 (L) and 10 (M) days post-germination. (N) Trichomes on primary leaf of seedling in (M). (O) Transcript abundance of *PAP85 *in Arabidopsis tissues. Gene expression data was extracted from the AtGenexpress Visualisation Tool () and Y-axis represents intensity of microarray signal in arbitrary units. Seed developmental stage approximates the time-scale of hours post-anthesis employed by Schmid et al. [42] as days post-anthesis (DPA) so as to enable more direct comparison between panels B-H and the published microarray data represented here.

Histochemical analysis of GUS activity in T2 plants revealed that *PAP85 *is predominantly expressed in the embryo and endosperm of developing seeds (Figure 6). In embryos, GUS activity began to be detectable at 5 days post anthesis (DPA; Figure 6D). Strong GUS activity persisted throughout subsequent stages of embryo development (Figure 6E–6H). The cotyledons and embryo axis appeared uniformly stained in all stages where GUS activity was detected, demonstrating *PAP85 *is broadly expressed in embryonic tissues. Strong and uniform GUS activity was also observed in the endosperm (Figure 6I). No GUS activity was detected in the embryos at 3 DPA or 4 DPA (Figure 6B and 6C) nor in floral tissues (Figure 6A). In young seedlings, GUS activity was strictly confined to the cotyledons, hypocotyl and root tip (Figure 6J–6N). GUS activity in the root tip persisted for 3–4 days after germination and was not detectable at later stages (Figure 6K and 6L). In expanded new leaves GUS activity was detected only in the trichomes; however, this activity was not detected in mature leaves. Collectively, these results precisely define the spatio-temporal expression pattern of the *PAP85 *promoter in Arabidopsis. These results are in good agreement with the transcript abundance profile of *PAP85 *(Figure 6O) obtained by querying the AtGenexpress Visualisation Tool () encompassing microarray analysis of an extensive collection of Arabidopsis tissues and developmental stages [42] and also with the *PAP85 *expression pattern during silique development reported by Parcy et al. [40]. Thus our system enables development of gene expression constructs that encode the regulatory sequences required to accurately represent the expression profile of the native gene. Our analysis of *PAP85 *also provides novel insight to its expression properties over the data currently publicly available with the higher-resolution reporter gene study presented here.

## Conclusion

Gene expression and phenotypes result from a combination of effects of classical upstream promoter elements and terminator sequences, as well as regulatory elements encoded by the 5'- and 3'-UTRs and introns [1,4-7]. Discovery of these effects may not be achieved when cDNAs and generic regulatory elements are used in gene expression studies or when only 5'-regulatory regions are used in reporter-gene studies. We anticipate that the system and plasmids described herein that employ a combination of Gateway and recombineering techniques should greatly expedite cloning and manipulation of gDNA fragments and enhance efforts in gene characterisation.

## Methods

### Bacterial strains and culture conditions

BACs F22P10 and F18C5 encoding the Arabidopsis genes *ABI3 *(At3g24650; GenBank: AJ002473) and *PAP85 *(At3g22640; GenBank: NM113163), respectively, were isolated from a library of the Col-0 ecotype in the vector pBeloBAC-Kan [33] and obtained from the Arabidopsis Biological Resource Centre in the *E. coli *DH10B host strain. *E. coli *EL250 is a derivative of DH10B and encodes the Red recombination functions linked to a temperature-sensitive promoter [27]. *E. coli *DB3.1 contains the *gyrA462 *mutation that enables propagation of Gateway vectors encoding the *ccdB *gene [21]. *A. tumefaciens *GV3101::pMP90 [43] was used to assess the functionality of plant transformation vectors. Where required, selection of plasmids was provided by culture in the presence of ampicillin (100 μg/ml), chloramphenicol (50 μg/ml), spectinomycin (80 μg/ml), tetracycline (12.5 μg/ml) or Zeocin™ (Invitrogen; 50 μg/ml).

### Construction of plasmids

The pENTR2B (Invitrogen) derivative pJM1 (GenBank: FJ391469) was created by amplifying the Tc^R ^marker from pACYC184 [44] using the PCR and primers Tc-5'-PmlNco and Tc-3'-PmlNsi to incorporate restriction sites. The nucleotide sequences of primers used to amplify the Tc^R ^marker, and other primers used in this study, are described in Table 1. The 1.6 kb amplicon encoding the Tc^R ^marker was digested with NcoI and NsiI and ligated into pENTR2B that had been digested with BspHI and NsiI to inactivate the resident Km^R ^marker.

The initial plant transformation vector pNML54 (GenBank: FJ410917) was created by amplifying the Sp^R ^marker from pEU904 [45] using the PCR and primers SpR-5'-HpaBspNhe and SpR-3'-Hpa to incorporate restriction sites. The 1.1 kb amplicon was digested with BspHI and HpaI and ligated into pCB301 [35] digested with BspHI and SspI to inactivate the resident Km^R ^marker. Selectable markers for use in plant transformations were then cloned into pNML54. A cassette encoding the EntCUP2 promoter [39] and polyadenylation signal from the nopaline synthase gene linked to the *NPTII *gene was obtained from pORE-O4 [46] by digestion with NcoI, treatment with the Klenow fragment of DNA polymerase and then digesting with NheI. The 1.7 kb cassette was ligated into pNML54 digested with EcoRV and SpeI resulting in pWY107 (GenBank: FJ410918) which can confer kanamycin resistance to transformed plants. The *NPTII *gene of pWY107 was excised by digestion with FseI and AscI and replaced with the *PAT *gene obtained from pORE-E3 [46] digested with the same enzymes resulting in pWY109 (GenBank: FJ410919) which can confer phosphinothricin-resistance to transformed plants.

Gateway Entry-type clones encoding GUS or GFP5 were created to link cloned regulatory signals to reporter genes by Gateway-based assembly. The GUS-encoding Entry-clone pTK185 (GenBank: FJ410921) was assembled by obtaining the GUS gene from pEntCUP5 [39] using BamHI and SpeI and ligating it to pTK172, a zeocin-resistant (Zeo^R^) derivative of pENTR1A (Invitrogen) digested with BamHI and XbaI. pTK172 (GenBank: FJ410920) was assembled by obtaining a Zeo^R ^marker from pEM7/Zeo (Invitrogen) using EcoRI and XhoI, making the ends blunt by treatment with T4 DNA polymerase, and ligating the cassette to pENTR1A digested with NsiI and NruI and also treated with T4 DNA polymerase. To assemble the GFP-encoding Entry clone pWY102 (GenBank: FJ410922), pAVA393 encoding GFP5 [47] was used as template in a PCR with the primers GFP-mu-5'Bam and GFP-mu-3'NotAvrBgl to incorporate convenient restriction sites. This process also repaired a mutation in the GFP5 gene encoded by pAVA393 by replacing missing coding information for a serine residue at position 2 and a glutamate residue at position 4 which when absent impaired the function of GFP as assessed by expression in *E. coli *(Rozwadowski and Yang, unpublished). The GFP5 amplicon was digested with BamHI and NotI and cloned into pENTR1A digested in a likewise manner. The function of this GFP5 cassette *in planta *was confirmed by transient assay in leaves of *Nicotiana benthamiana *(Rozwadowski and Pyne, unpublished). Constructs linking the GUS or GFP genes with cloned transcription regulatory regions were created using the LR Clonase (Invitrogen) reaction following the directions of the manufacturer.

### Recombineering Procedures

To rescue (i.e. subclone) the *ABI3 *coding region from BAC F22P10 (GenBank: B96472 and B96473), pJM1 was used as template in a PCR with Elongase Enzyme Mix (Invitrogen) and the primers ABI3-L1-Rescue-5'-CDS and ABI3-L2-Rescue-3'-CDS. The resultant 3.5 kb pJM1 amplicon was gel-purified and approximately 300 ng was electroporated into *E. coli *EL250/F22P10 competent cells prepared as per Lee et al. (2001) to accumulate Red homologous recombination proteins, then the cells were plated in the presence of tetracycline. After 2 d at 30°C, colonies were assessed for the correctly rescued *ABI3 *coding region by PCR using the primers ENTRY-L1 combined with ABI3-5'-CDS-Rescue-3'-Test and ENTRY-L2 combined with ABI3-3'-CDS-Rescue-5'-Test.

To rescue the *PAP85 *coding and regulatory regions from BAC F18C5 (GenBank: B22277 and B22278), pWY109 was first digested with SacI then used as template in a PCR with Elongase Enzyme Mix (Invitrogen) and the primers PAP85-Rescue-5'-Prom combined with PAP85-Rescue-3'-Term. The 5.6 kb pWY109 amplicon was gel purified and approximately 300 ng was transformed into *E. coli *EL250/F18C5 competent cells expressing Red homologous recombination proteins, then the cells were plated in the presence of spectinomycin. After 2 d at 30°C, colonies were assessed for the correctly rescued *PAP85 *gene by PCR using the primers pCB-RB combined with PAP85-Prom-Rescue-3'-Test and tCUP-Out combined with PAP85-Term-Rescue-5'-Test.

To replace the coding region of *PAP85 *with the Gateway RfA-Destination (Invitrogen) cassette by recombineering, pWY189 was first cotransformed into *E. coli *DB3.1 with pKD46 [41]. The RfA-Destination cassette encoding a Cm^R ^marker and *ccdB *flanked by attR1 and attR2 was amplified by PCR to incorporate appropriate homology regions to enable its recombination-mediated replacement of the *PAP85 *coding region in pWY189. pMW138-1 encoding the RfA-Destination cassette cloned into the EcoRV site of pBluescript KS- (Stratagene) was first digested with AhdI then used as template in a PCR with primers PAP85-Integ-Dest-5' and PAP85-Integ-Dest-3'. The 1.8 kb RfA-cassette amplicon was gel purified and approximately 300 ng was electroporated into *E. coli *DB3.1/pKD46/pWY189 cells prepared to accumulate Red recombinase proteins by induction with arabinose [41], then plated in the presence of spectinomycin and chloramphenicol. After 2 d at 30°C, candidate clones were assessed for correct integration of the RfA-Destination cassette by PCR using the primers PAP85-Dest-Integ-5'-Test combined with Cm-5'-AS and PAP85-Dest-Integ-3'-Test combined with C1. The strain was cured of pKD46 by culturing at 42°C as per Datsenkeo and Wanner [41], and screened for Sp^R^, ampicillin-sensitive (Ap^S^) colonies; these isolates possessed both pWY190 and the parental pWY189. pWY190 was purified from pWY189 by digesting a plasmid preparation with BlpI, which uniquely cleaves pWY189, and then transforming the mixture into *E. coli *DB3.1 and plating in the presence of spectinomycin. Colonies were screened by PCR for the presence of pWY190 using primers PAP85-Dest-Integ-3'-Test combined with C1 and the absence of pWY189 using primers PAP85-Dest-Integ-3'-Test combined with PAP85-S1. Restriction digestion with EcoRI confirmed the integrity of pWY190 and absence of pWY189.

### Construction and analysis of *PAP85::GUS *reporter construct

The GUS gene of pTK185 was transferred into pWY190 following standard Gateway procedures resulting in the *PAP85::GUS *reporter construct designated as pSW27. This construct was introduced into *Agrobacterium tumefaciens *GV3101::pMP90 [43]. *Arabidopsis thaliana *ecotype Columbia plants were transformed by the floral dip method [48]. Several independent transgenic lines were identified by selecting for phosphinothricin resistance encoded by the T-DNA. GUS activity was visualized by histochemical staining [49]. Tissues were cleared of cholorophyll by immersing in 90% ethanol, and photographed using a stereomicroscope.

## Authors' contributions

KR conceived, designed and coordinated the study, and drafted the manuscript. WY developed and established protocols, assembled constructs and aided in manuscript preparation. SK characterized transgenic plant lines and aided in manuscript preparation. All authors read and approved the final manuscript.
